# Orbital Cellulitis Secondary to Dental Abscess in Children

**DOI:** 10.7759/cureus.14392

**Published:** 2021-04-09

**Authors:** Huwaina Abdul Satar, Azhany Yaakub, Norasnieda Md Shukri, Liza Sharmini Ahmad Tajudin

**Affiliations:** 1 Ophthalmology Clinic, Hospital Universiti Sains Malaysia, Universiti Sains Malaysia, Kelantan, MYS; 2 Department of Ophthalmology and Visual Science, School of Medical Sciences, Universiti Sains Malaysia, Kelantan, MYS; 3 Department of Otorhinolaryngology, School of Medical Sciences, Universiti Sains Malaysia, Kelantan, MYS

**Keywords:** orbital cellulitis, dental caries, gum abscess, children.

## Abstract

Paediatric orbital cellulitis is a potential sight- and life-threatening condition. It is a serious infection in children that can result in significant complications, including blindness, cavernous sinus thrombosis, cerebral venous sinus thrombosis, meningitis, subdural empyema, and brain abscess. Of the patients with orbital cellulitis, 17% died from meningitis, and 20% of the survivors had permanent loss of vision. Therefore, the potential for sight- and life-threatening complications makes prompt diagnosis and early treatment very important. We report here a case of a two-year-old girl who presented with a three-day history of left periorbital swelling, preceded by left upper perioral swelling that extended upward to the left cheek and left lower lid and was associated with low-grade fever. The patient had been admitted and was treated as having left preseptal with facial cellulitis; the patient was started on intravenous amoxicillin/clavulanic acid (200 mg three times per day dose), and chloramphenicol ointment was applied to the periorbital area. On day 3, the condition worsened, and dental examination showed multiple dental caries, upper gum swelling and abscess, and mobility of teeth 61 and 62 (two baby teeth). Contrast-enhanced computed tomography (CECT) of the orbit, paranasal, and brain showed a left periosteal abscess collection extending to the inferomedial region of the orbit. Examination and tooth extraction were performed under general anesthesia. The intraoperative results showed the presence of a left upper gum abscess, which was possibly the primary source of infection. Clinical improvement was observed postoperatively. Orbital cellulitis can be a complication of a dental abscess. This case emphasizes the importance of primary tooth care in children. A lack of care can result in fatal complications.

## Introduction

Paediatric orbital cellulitis is a sight- and life-threatening condition that can lead to devastating complications including blindness, cavernous and cerebral venous sinus thrombosis, meningitis, subdural empyema, and brain abscesses. The most common risk factors of orbital cellulitis are sinusitis and trauma [1]. Other risk factors include dental abscesses, middle ear infections, and intracranial infections [2]. The most common organisms responsible for pediatric orbital cellulitis are Staphylococcus followed by Streptococcus species and *Haemophilus influenza* [3]. Orbital cellulitis is a rare but serious sequelae complication of dental infection [4]. Dental caries is one of the most common dental problems affecting children. Dental caries and infection are important oral health problems amongst school children aged 6-18 years old in Malaysia, showing a distinct variation in three racial groups [5]. Untreated dental caries in children may lead to rare complications, such as orbital cellulitis, orbital abscess with impairment, or loss of vision. Left untreated, the condition can progress to blindness, cavernous sinus thrombosis, meningitis, subdural empyema, brain abscesses, and death. Awareness of the possible spread of odontogenic infection to the orbit is extremely important, so vigorous treatment may start as early as possible. This article was previously presented as a meeting abstract and poster at the 2019 COCS Conjoint Ophthalmology Scientific Conference on September 13, 2019.

## Case presentation

A two-year-old girl presented with a three-day history of progressive left periorbital swelling associated with low-grade fever. The swelling first appeared on the left upper perioral region, and extended upward to the left cheek and left lower lid. However, no eye redness was observed, nor was there any watery, eye, ear, or nasal discharge. There was no history of trauma.

At presentation, the patient was active, had stable vital signs, and was febrile with a temperature of 38 °C. Ocular examination revealed left eyelid redness and a firm left periorbital swelling without bite marks or discharge from the upper and lower punctum. There was no proptosis and no relative afferent pupillary defect. The conjunctiva was white, with no chemosis. Extraocular muscle movements, anterior segments and dilated fundus examination were normal. However, the eye swab culture, blood culture and sensitivity were negative.

**Figure 1:**
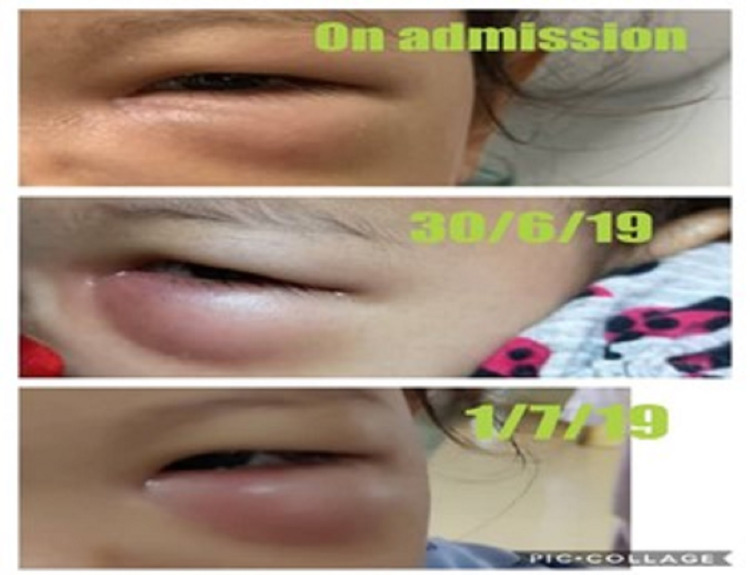
The progression of the left periorbital swelling. The condition worsened on day 3 after admission.

**Figure 2:**
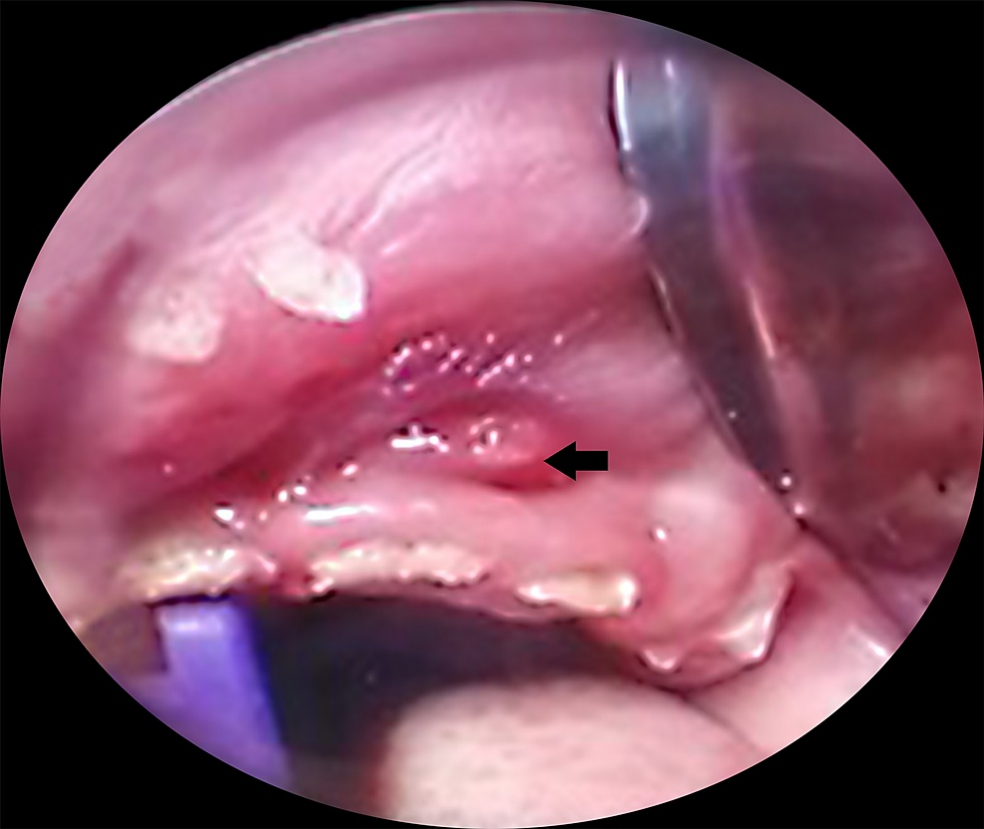
Dental examination under general anaesthesia showed multiple dental caries and upper gum abscess (arrow).

**Figure 3:**
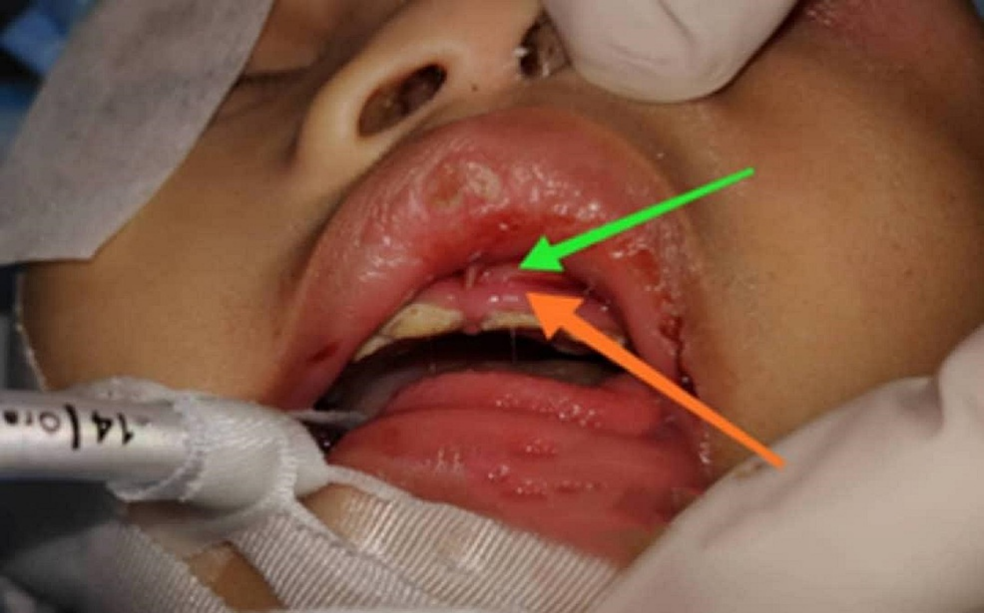
Upper gum swelling with abscess collection. The orange arrow indicates the gum swelling and the green arrow indicates the abscess collection.

The patient was admitted with a diagnosis of left preseptal cellulitis and started on intravenous amoxicillin/clavulanic acid (200 mg three times a day), and chloramphenicol ointment was applied to the periorbital area. On day 3 of intravenous antibiotic administration, the swelling increased in size, involved the upper lid, and became generalised periorbital swelling (Figure 1). A dental examination showed multiple dental caries, upper gum swelling and abscess, and mobility of teeth 61 and 62 (two baby teeth; Figures 2 and 3). Nasal scope by the otorhinolaryngology team revealed normal findings.

**Figure 4:**
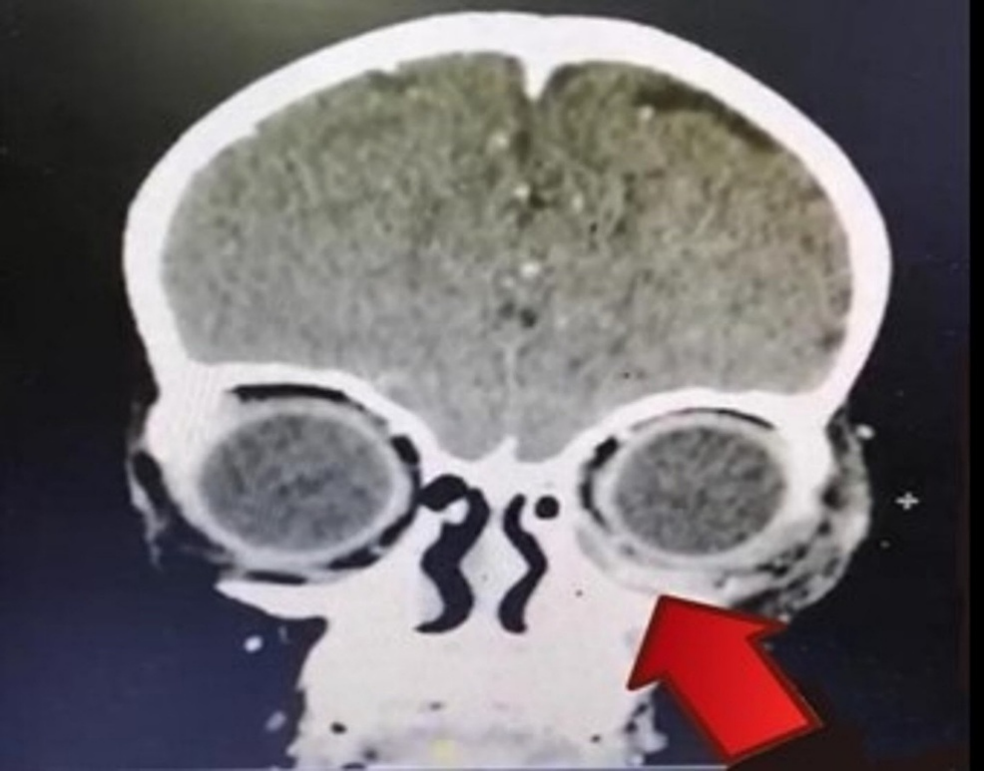
The left periosteal abscess collection extending into the inferomedial aspect of the left orbit.

Contrast-enhanced computed tomography (CECT) of the orbit, paranasal, and brain showed a left periosteal abscess collection extending into the inferomedial aspect of the left orbit (Figure 4), left maxillary, and ethmoidal sinuses. However, no cavernous sinus thrombosis or intracranial extension was observed. Examination and tooth extraction under general anaesthesia were performed, and the intraoperative results showed the presence of a left upper gum abscess, which was the possible primary source of infection. The culture of the gum abscess was taken and no organism was isolated. The patient completed a one-week course of intravenous amoxicillin/clavulanic acid and metronidazole. Clinical improvement was observed with a reduction in left upper lid swelling and an afebrile reading prior to discharge.

## Discussion

Orbital cellulitis is a serious infection in children which can result in significant complications, including blindness, cavernous sinus thrombosis, meningitis, subdural empyema, and brain abscess [6,7]. Periorbital infection is classified as either preseptal or orbital, depending on its restriction by the anatomical layer of fascia extending vertically from the orbital rim to the tarsal plates, known as the orbital septum. This orbital septum prevents many preseptal infections from spreading to the deeper orbital and retro-orbital structures. Preseptal cellulitis occurs anterior to the orbital septum and can result from trauma, contiguous infection, or primary bacteraemia among infants [5]. In orbital cellulitis, the infection is localized posterior to the orbital septum and usually occurs as a complication of acute or chronic sinusitis [6]. Both conditions are more common in children than in adults, and preseptal cellulitis is nearly three times more common than orbital cellulitis. The infection may be collected under the periosteum and lead to a subperiosteal abscess or intraorbital abscess formed secondary to progressive and localized cellulitis [6].

In the literature, the most commonly reported cause of orbital cellulitis or abscess in children is maxillary or ethmoidal sinusitis. However, the distant spread of infection from a dental abscess has also been reported, even though it is rare [8]. Maxillary teeth infections are known to involve the maxillary sinus, inferior orbital fissure, and reach orbit, or they can perforate through the eyelid and preseptal space and enter the orbit [9]. In the present case, the infection started in the upper primary teeth, involved an upper gum abscess, the maxillary sinus, and the orbit, and perforated the thin orbital floor/inferior orbital fissure. The primary source of infection was traced back to the area of teeth 61 and 62, which showed an initial swelling that started at the upper perioral region. It subsequently became localised and extended posteriorly into the sinuses and orbit. It also possibly perforated the orbital septum anteriorly and collected at the periosteal region, as there was a periosteal abscess collection that extended into the inferomedial aspect of the left orbit.

In acute orbital infection, swelling of the eyelids due to reactive oedema or cellulitis often makes adequate physical examination of the eye difficult or impossible. Precise delineation of the source and extent of the infection is important for adequate and correct treatment [10]. Imaging studies are of critical importance in defining the extent and nature of orbital inflammation and in determining appropriate management. CECT provides an invaluable source of information about these infections, and the images can be examined for the presence of an orbital abscess [11]. As in our case, CECT scans of the orbit, paranasal, and brain were performed after the patient’s condition was noted to be worsening on day 3 after admission. CECT images showed a left periosteal abscess collection extending into the inferomedial aspect of the left orbit, left maxillary, and ethmoidal sinuses, but no intracranial extension or cavernous sinus thrombosis. This infection spread upward from the primary site of the dental abscess to the maxillary sinuses and extended into the orbit.

Optimal management of patients with orbital cellulitis depends on how accurately the disease is classified and whether appropriate antibiotics and surgery are used to treat the disease. The disease classification follows that outlined by Schramm et al. and includes preseptal cellulitis, orbital cellulitis, subperiosteal abscess, orbital abscess, and cavernous sinus thrombosis [12]. This classification is important because it emphasizes the possibility of death due to cavernous sinus thrombosis and intracranial abscess. Early identification of the primary source of infection and multidisciplinary management is critical for adequate and appropriate treatment. As demonstrated in this case, early presentation, appropriate intravenous antibiotics, and early referral with co-management by an otorhinolaryngologist (ORL) and the dental team ensured optimal care for this patient.

## Conclusions

A clinical diagnosis of orbital cellulitis in children is sometimes difficult, but obtaining a detailed history, performing a thorough and complete physical examination, and conducting a detailed review of laboratory and imaging modalities may guide healthcare providers towards the correct diagnosis. Multidisciplinary referral and management are important in dealing with paediatric cases to ensure optimal management and care. This case also emphasizes the importance of primary tooth care in children; a lack of care can lead to the distant spread of infection and associated complications, such as loss of vision and death.
